# Potentially Toxic Elements in Surface Sediments of Black-Necked Crane Habitats: A Case Study of Plateau Lakes in Shangri-La, Yunnan, China

**DOI:** 10.3390/ani16193012

**Published:** 2026-09-24

**Authors:** Mumo Li, Zhen Peng, Zhenghua He, Shilong Liu, Xu Li, Hua Yang, Ruobing Han

**Affiliations:** 1 Key Laboratory for Conserving Wildlife with Small Populations in Yunnan, College of Forestry, Southwest Forestry University, Kunming 650224, China; 2 Potatso National Park Administration, Diqing 674400, China; 3 Dali Branch of the Yunnan Institute of Forest Inventory and Planning, Dali 671000, China

**Keywords:** plateau lakes, black-necked crane, heavy metals, Potatso National Park

## Abstract

Plateau wetlands provide important wintering habitats for migratory waterbirds, making the assessment of environmental contamination relevant to their conservation. Characterizing potentially toxic elements in lake sediments provides a basis for identifying contamination patterns and prioritizing environmental monitoring in these habitats. Here, we assessed potentially toxic element contamination in surface sediments and potential ecological risks in three plateau lakes supporting wintering black-necked cranes (*Grus nigricollis*) in Shangri-La, Yunnan, China. In January 2025, 131 composite sediment samples (0–20 cm) were collected from Bitahai, Shudu, and Napahai lakes. Concentrations of Cu, Zn, Pb, As, Hg, and Cd were determined by inductively coupled plasma mass spectrometry. Contamination and potential ecological risks were evaluated using the geo-accumulation index (*I_geo_*) and potential ecological risk index (PERI), respectively. Potential sources were investigated using EPA PMF 5.0. Sediments were generally unpolluted to slightly polluted. Mean PERI values were 65.2, 62.7, and 76.5, respectively, indicating low potential ecological risk, with Cd and Hg contributing most. PMF resolved four factors tentatively associated with traffic/domestic emissions, agricultural/combustion inputs, localized inputs, and natural/regional sources. These findings provide a baseline for sediment monitoring and environmental management of black-necked crane wintering habitats, with particular attention to Cd, Hg, and As.

## Simple Summary

The black-necked crane (*Grus nigricollis*) is a flagship species that winters in the plateau lakes of Shangri-La, Yunnan Province, specifically Bitahai Lake, Shudu Lake, and Napahai Lake, which serve as critical foraging grounds and refuges during the cold season. However, surrounding human activities, including farming, road traffic, coal burning, and tourism, may release harmful metals into these lakes, posing potential health risks to the cranes and other waterbirds. This study assessed the concentrations, sources, and ecological risks of potentially toxic elements in the surface sediments of all three lakes. Heavy metal concentrations exceeded local soil background levels at multiple sampling points. Notably, Napahai Lake, which is outside the national park, exhibited a relatively higher ecological risk than the two lakes inside the park (Bitahai Lake and Shudu Lake). Source apportionment indicated that traffic and household waste were the main contributors of Cu, Zn, and Pb; farming and coal burning contributed to Cd and Hg; while agriculture and local discharge points were responsible for As. The results offer valuable guidance for safeguarding the wintering grounds of the black-necked crane, supporting the conservation of this vulnerable species, and informing the management of other fragile high-altitude lake ecosystems that shelter migratory waterbirds.

## 1. Introduction

An important strategy for maintaining regional biodiversity is the conservation of flagship or umbrella species, which in turn contributes to the protection of the entire ecosystem [1,2,3]. The black-necked crane (*Grus nigricollis*), serving as both a flagship species and an environmental indicator of highland cold wetland ecosystems, plays an indispensable role in sustaining biodiversity within plateau wetlands [4,5]. It is the only crane species worldwide that inhabits plateau environments throughout its entire life cycle [6,7], and is currently listed as Near Threatened (NT) on the IUCN Red List [8,9]. During the breeding season, black-necked cranes occupy wetlands on the Qinghai–Tibet Plateau, subsequently migrating to the Yunnan-Guizhou Plateau for wintering [10]. At their wintering sites, these cranes preferentially utilize habitats such as marshes, lakeside meadows, shallow flats, open warm-water rivers, and sheltered leeward wetlands [11,12]. The plateau lakes of Shangri-La, located in the southeastern Qinghai–Tibet Plateau and northwestern Yunnan Province, including Bitahai and Shudu Lakes within Potatso National Park and Napahai Lake outside its boundaries. These wetlands provide wintering habitat for black-necked cranes and are therefore relevant to regional habitat conservation. However, in recent years, the construction of irrigation projects and drainage systems, along with high-intensity grazing, has caused certain damage to wetlands, thereby threatening the survival of the black-necked crane. This phenomenon has been proved in the Yarlung Tsangpo River basin in Tibet [13], and in the Napahai Wetland and the Caohai Wetland lake areas [14]. Therefore, protecting the wintering habitats of the black-necked crane is critically important, as it not only concerns the survival of this rare species but also serves as a key factor in maintaining the health of plateau wetland ecosystems and regional ecological balance.

Plateau lakes are pivotal components of alpine ecosystems that mediate hydrological regulation. Compared with lowland lakes, plateau lakes generally exhibit greater ecological fragility and environmental sensitivity [15] making them responses to environmental changes more pronounced and complex [16,17]. Among various environmental stressors, contamination by potentially toxic elements has attracted particular concern because these elements persist in the environment and may exert toxic effects when present in bioavailable forms [18]. Potentially toxic elements including Cd, Hg, As, Pb, Cu, and Zn can accumulate in lake sediments and may be remobilized as physicochemical conditions change [19,20]. Sediment measurements therefore provide information on contaminant accumulation and a basis for screening potential ecological concerns. For wintering waterbirds such as the black-necked crane, foraging in wetlands may provide opportunities for exposure to sediment-associated contaminants through incidental sediment ingestion or consumption of contaminated food [21]. Thus, investigating potentially toxic element contamination in sediments of plateau lakes that serve as wintering habitats for the black-necked crane provides an important scientific basis for the protection of fragile plateau lake ecosystems and for identifying priorities for further exposure assessment in wintering waterbirds such as the black-necked crane.

The six potentially toxic elements considered here differ in their biological functions and toxicological significance. Cd, Hg, As, and Pb are of particular toxicological concern because exposure to these elements can adversely affect multiple organ systems, whereas Cu and Zn are essential trace elements but can be toxic at excessive exposure levels [22]. Previous studies have identified Cd, Hg, and As as important contributors to potential ecological risk in lake sediments, as exemplified by long-term sedimentary records from western Taihu Lake and southwestern alpine lakes, and by surface sediment investigations in Dongping Lake and the Dianchi Lake basin on the Yunnan Plateau [23,24]. In Caohai, another plateau wetland used by black-necked cranes, sediment investigations have documented all six elements and reported contamination by Cd, Hg, and Zn [19]. A longer-term assessment also reported mean sediment concentrations of As, Cd, Hg, Pb, and Zn above regional background values [25]. Moreover, through trophic transfer and biomagnification in aquatic food webs, heavy metals can progressively threaten fish and waterbird populations and ultimately elevate human exposure risks [26,27]. However, previous research on potentially toxic elements in black-necked crane habitats has focused mainly on Caohai Wetland [28,29,30,31], revealing that black-necked cranes are suffering from multi-element heavy metal contamination and investigating potential exposure pathways. Limited research has been conducted on the plateau lakes in Shangri-La, Yunnan, China, another important wintering ground for the black-necked crane. Investigating the levels of potentially toxic element contamination across different habitats of the black-necked crane is essential for developing effective conservation measures, scientific management strategies, and protecting the habitats upon which this species depends.

Herein, we measured the concentrations of six potentially toxic elements (Cu, Zn, As, Cd, Hg, and Pb) in surface sediments of three plateau lakes (Shudu Lake, Bitahai Lake, and Napahai Lake) in Shangri-La, Yunnan, China. These lakes serve as important wintering habitats for the black-necked crane, and their ecological risks were also assessed. Specifically, the objectives of this study were to: (1) assess pollution levels and ecological risks in the black-necked crane habitats, and (2) identify pollution sources and conduct source-specific risk assessment. The results offer a scientific foundation for preserving the ecological integrity of Potatso National Park, and provide an important reference basis for protecting fragile plateau lake ecosystems and for developing effective conservation and management strategies for the black-necked crane and its habitats.

## 2. Materials and Methods

### 2.1. Study Area

Bitahai Lake, Shudu Lake, and Napahai Lake are located in Shangri-La on the northwestern Yunnan Plateau, adjacent to the three parallel rivers world natural heritage area (Figure S1). As representative plateau lakes on the eastern margin of the Tibetan Plateau, they share similar climatic conditions (characterized by an alpine mountain climate), altitudinal gradients, and vegetation types (forming a coniferous forest-meadow-lake mosaic system), and serve as wintering habitats for migratory birds such as the black-necked crane. However, they exhibit significant differences in watershed land use, intensity of human activity, and potential pollution sources. Both Bitahai Lake and Shudu Lake are situated within Potatso National Park. Bitahai Lake is surrounded by well-preserved cold-temperate coniferous forest and experiences minimal external anthropogenic disturbance [32,33]. In contrast, Shudu Lake is a tectonic fault depression glacial relic lake characterized by a small catchment area and short flow paths, rendering it highly sensitive to lithologic weathering and atmospheric deposition inputs [34,35]. Outside the national park, Napahai Lake (also in Shangri-La) is an open lake bordered by agricultural fields, grazing areas, and villages, exposing it to greater anthropogenic influence.

### 2.2. Sample Collection

In January 2025, a total of 131 surface sediment samples were collected from three plateau lakes (Bitahai, Shudu, and Napahai Lakes) in Shangri-La, with 35, 57, and 39 sampling sites, respectively. Specifically, based on previous monitoring records of Potatso National Park and field observations, the main foraging waters of the black-necked crane and their adjacent shoreline areas were selected as sampling sites. At each site, a soil sampler was used to collect 0–20 cm surface samples. A sampling area of approximately 15 m × 15 m was delineated, and five subsamples were collected using the five-point sampling method (four corners and the center) and thoroughly mixed to form one composite sample. Each composite sample was placed into a resealable plastic bag, labeled with the sampling ID, date, and location, and then transported to the laboratory. The sampling process adhered to the principles of representativeness, uniformity, and typicality.

### 2.3. Sample Processing

Prior to elemental analysis, the air-dried sediment samples were processed as follows. Extraneous materials such as rock fragments, gravels, and plant residues were manually removed. To prevent cross-contamination, metal tools were avoided and all equipment was thoroughly cleaned between samples.

For Cu, Cd, Zn, As, Hg, and Pb determination, sediment aliquots of 0.05 g, weighed to the nearest 0.0001 g, were placed in digestion vessels, treated with 5 mL of concentrated HNO_3_, and left overnight. Subsequently, 2 mL of H_2_O_2_ and 1 mL of HF were added. The vessels were sealed in stainless-steel jackets and heated in a thermostatically controlled oven at 150–170 °C for 4 h. After cooling, the jackets were carefully opened, and the inner digestion vessels were removed and heated on a hot plate at 160 °C for 30 min. The digests were subsequently cooled and diluted to 25 mL with 1% HNO_3_. Cu, Cd, Zn, and Pb concentrations were determined using inductively coupled plasma mass spectrometry (ICP-MS; NexION 1000, PerkinElmer, Shelton, CT, USA). Moreover, the ICP-MS instrument was tuned in KED QID collision mode, with Rh used as the internal standard. Ar was supplied at 90 psi, and the plasma and nebulizer gas flow rates were set to 17 and 0.97 L min^−1^, respectively. The spray chamber temperature was maintained at 3 °C. Quantitative analysis was performed using peak-hopping acquisition. Sample uptake, stabilization, and rinsing times were 50, 15, and 50 s, with corresponding peristaltic pump speeds of 42, 35, and 42 rpm, respectively [36,37].

The metal concentrations in the digestion solution measured by the instrument were corrected using the average concentration of the procedural blank from the same batch. The concentrations of heavy metals in the sediment were then calculated based on the sample mass and the final constant volume, using the following formula:

$$C=\frac{(C_{m}-C_{b})\times V}{1000\times m}$$

where *C* is the concentration of heavy metals in the sediment (mg/kg), *C_m_* is the metal concentration in the digestion solution measured by the instrument (μg/L), *C_b_* is the average concentration of the corresponding metal in the procedural blank from the same batch (μg/L), *V* is the final constant volume of the digestion solution (mL), and *m* is the dry weight of the sediment sample (g).

The certified reference material GBW07407 was used to evaluate analytical accuracy. The average recoveries of Cu, Zn, As, Cd, Hg, and Pb were 98.52%, 102.10%, 94.11%, 107.33%, 101.45%, and 100.59%, respectively. Parallel samples were used to evaluate analytical precision, and the relative percent deviation (RPD) of all parallel samples was below 25%, with a maximum value of 20.74%. The internal standard recovery ranged from 85.1% to 105.9%. The method detection limits for Cu, Zn, As, Cd, Hg, and Pb were 0.05, 0.5, 0.002, 0.002, 0.001, and 0.02 μg/g, respectively. All calibration curves met the requirement of R^2^ ≥ 0.995.

### 2.4. Statistical Analysis

To characterize the concentration patterns of heavy metals in lake sediments, the minimum, maximum, median, mean, standard deviation (SD), and coefficient of variation (CV) were calculated. Normality of the data was assessed using the Shapiro–Wilk test, and homogeneity of variance was evaluated using Levene’s test. Since the heavy metal concentration data did not follow a normal distribution, the non-parametric Kruskal–Wallis H test (degrees of freedom, df = 2) was employed to assess overall differences in heavy metal concentrations among the three lakes (Shudu Lake, Bitahai Lake, and Napahai Lake). When significant overall differences were detected, Mann–Whitney U tests were conducted for post-hoc pairwise comparisons. To control for the Type I error rate arising from multiple testing, *p* values were adjusted using the Holm-Bonferroni correction. A corrected *p* value of less than 0.05 was considered statistically significant.

Correlations among heavy metals were assessed using Spearman’s rank correlation coefficients, with statistical significance set at *p* < 0.05. To identify co-variation patterns and potential common sources among the metals, PCA was performed on standardized data. Variables with absolute loadings exceeding 0.5 were considered significant contributors to the corresponding principal components [38].

Data management and statistical analyses were conducted using Microsoft Excel 2019 and R software (V4.4.2), respectively. Statistical graphs were generated with Origin 2021. Source apportionment was performed with the EPA PMF 5.0 model.

#### 2.4.1. Ecological Risk Assessment

The potential ecological risks posed by heavy metals in the sediments were assessed using the single-element potential ecological risk factor ($E_{r}^{i}$) and the PERI [39]. First, the contamination factor for each metal (i) was calculated as follows:

$$C_{r}^{i}=\frac{C_{i}}{B_{i}}$$

where (C_i_) is the measured concentration of metal (i) in the sediment, and B_i_ is the corresponding local geochemical background value. The single-element potential ecological risk factor was subsequently calculated using the equation:

$$E_{r}^{i}=T_{r}^{i}\;\times \;C_{r}^{i}$$

where $T_{r}^{i}$ represents the toxic-response factor for metal i. The target metals (Cd, Hg, As, Pb, Cu, and Zn) and the risk classification criteria were adopted from the original methodology [39]. The comprehensive potential ecological risk index is given by:

$$PERI=\sum E_{r}^{i}$$

Generally, the risk level based on $E_{r}^{i}$ is categorized as low (<40), moderate (40–80), considerable (80–160), high (160–320), and very high (>320). For the comprehensive PERI, the risk levels are categorized as low (<150), moderate (150–300), considerable (300–600), and very high (>600) [39]. This index system has been widely applied to assess the potential ecological risks of heavy metals in various aquatic and terrestrial environments, including rivers, lakes, and soils [40,41].

#### 2.4.2. Risk Assessment

The extent of heavy metal pollution in the sediments was assessed using the *I_geo_*. The *I_geo_* is defined as the logarithm of the ratio of the measured concentration of a heavy metal to its local geochemical background value. This approach accounts for both natural background levels and anthropogenic influences, thereby enabling a quantitative evaluation of heavy metal accumulation in sediments and its associated potential environmental impacts. The corresponding classification criteria for *I_geo_* values are provided in Table 1 [42]. In this study, the local soil background values were used as the reference for each metal [43]. The *I_geo_* was calculated using Equation (1).

(1) $$I_{geo}\;=\;{log}_{2}\frac{C_{n}}{{1.5B}_{n}}$$

where *I_geo_* is the geo-accumulation index, C_n_ represents the measured concentration of the heavy metal (mg·kg^−1^), and B_n_ is the corresponding local background value of the heavy metal (mg·kg^−1^).

#### 2.4.3. PMF Source Apportionment Method

The PMF model was employed to identify and quantify the contributions of major heavy metals sources in the sediments. The model was implemented using EPA PMF 5.0 software [44]. Potential sources considered included natural parent materials, agriculture activities, traffic-related emissions, and atmospheric deposition.

In the PMF framework, the measured concentration data are structured into a data matrix X of dimensions i × j. The calculation formula is as follows [45,46]:

$$X_{ij}=\sum_{k=1}^{p}g_{ik}f_{kj}+e_{ij}$$

$$Q=\sum_{i=1}^{n}\sum_{j=1}^{m}{\left(\frac{X_{ij}-\sum_{k=1}^{p}g_{ik}f_{kj}}{u_{ij}}\right)}^{2}=\sum_{i=1}^{n}\sum_{j=1}^{m}{\left(\frac{e_{ij}}{u_{ij}}\right)}^{2}$$

$$u_{ij}=\frac{5}{6}\times C_{mdl}$$

$$u_{ij}=\sqrt{{\left(E_{f}\times C_{ij}\right)}^{2}+{\left(0.5\times C_{mdl}\right)}^{2}}$$

Here, *u*_*ij*_ is the measurement uncertainty for component *j* in sample *i* (µg·m^−3^), *C_mdl_* is the method detection limit, *C_ij_* is the measured mass concentration, and $E_{f}$ is the error fraction.

## 3. Results

### 3.1. Potentially Toxic Elements Contamination Characteristics in Surface Sediments of Plateau Lakes in Shangri-La

The mean concentrations of Cu, Zn, As, Cd, Hg, and Pb differed among the three lakes. Shudu Lake exhibited values of 21.21 ± 7.85 μg/g, 53.56 ± 21.81 μg/g, 9.13 ± 3.27 μg/g, 0.23 ± 0.11 μg/g, 0.031 ± 002 μg/g, and 23.70 ± 6.71 μg/g, respectively. In Bitahai Lake, the corresponding averages were 36.15 ± 10.06 μg/g (Cu), 70.69 ± 14.96 μg/g (Zn), 10.67 ± 4.42 μg/g (As), 0.19 ± 0.09 μg/g (Cd), 0.038 ± 0.042 μg/g (Hg), and 23.11 ± 9.2 μg/g (Pb). For Napahai Lake, the levels were 29.59 ± 10.26 μg/g (Cu), 65.27 ± 19.63 μg/g (Zn), 15.53 ± 5.58 μg/g (As), 0.31 ± 0.17 μg/g (Cd), 0.0285 ± 0.001 μg/g (Hg), and 17.83 ± 3.75 μg/g (Pb) (Table S1). All values are presented as mean ± standard deviation (SD).

The background values of Cu, Zn, As, Cd, Hg, and Pb in soils of Yunnan are 46.3, 89.7, 18.4, 0.22, 0.06, and 40.6 μg/g, respectively. Heavy metal concentrations exceeding the soil background values were observed at a number of sampling sites. For Cu, only 1 site exceeded the background value in Shudu Lake and 7 sites in Bitahai Lake, while no sites exceeded the background value in Napahai Lake. For Zn, the exceedance was observed at 2 sites in Shudu Lake, 3 sites in Bitahai Lake, and 5 sites in Napahai Lake. For As, 1 site in Shudu Lake, 2 sites in Bitahai Lake, and 13 sites in Napahai Lake exceeded the background value. Cd showed the most widespread exceedance, with 24 sites in Shudu Lake, 10 sites in Bitahai Lake, and 23 sites in Napahai Lake. For Pb, 1 site in Shudu Lake and 3 sites in Bitahai Lake exceeded the background value, whereas no sites exceeded it in Napahai Lake. No sites exceeded the background value for Hg in any of the three lakes. In addition, the concentrations of Cu, Zn, As, Cd, and Pb in Shudu Lake and Bitahai Lake were moderately variable, whereas Hg in Bitahai Lake was highly variable. In Napahai Lake, Cu, Zn, As, and Pb were moderately variable, but Cd was highly variable (Table S1).

Given the high variability of Cd in Napahai Lake and Hg in Bitahai Lake, along with their exceedance patterns, we mapped the exceedance locations of Cd, Hg, and As in relation to nearby human activity features to further clarify the spatial characteristics of exceedance sites (Figures S2–S4). In Napahai Lake, Cd exceedance sites were mainly distributed near roads and guesthouse areas, while the co-occurrence of Cd and As exceedances was observed near villages and pasture zones. In Shudu Lake, four sampling sites showed exceedances and were located near roads, a pier, pasture, and inflow-affected areas. In Bitahai Lake, the localized co-exceedance site of Cd and Hg was situated near a pier.

### 3.2. Spatial Differences in Potentially Toxic Elements in Surface Sediments of the Three Plateau Lakes

The six heavy metals displayed distinct spatial distribution patterns and pronounced inter-lake variability among the three plateau lakes (Figure 1). Specifically, Napahai Lake exhibited the highest concentrations of Cd and As, whereas the lowest concentrations of Hg and Pb were observed, with relatively confined spatial distributions. Bitahai Lake generally showed higher Zn levels, while Shudu Lake was characterized by elevated Pb, relatively higher Hg, and a broader dispersion of Zn concentrations, implying greater within-lake heterogeneity for certain metals. Consistent with these patterns, significant inter-lake differences in the concentrations of Cu, As, Hg, and Pb were observed between Napahai Lake and Shudu Lake. Notable differences in As, Hg, and Pb were also evident between Bitahai Lake and Napahai Lake. Moreover, significant differences in Cu and Zn concentrations were identified between Bitahai Lake and Shudu Lake (Figure 1).

### 3.3. Ecological Risk Assessment of Potentially Toxic Elements in Surface Sediments

The *I_geo_* results indicated higher *I_geo_* values for Cd in Napahai Lake and elevated Hg levels at specific sites in Bitahai Lake, whereas Hg in Shudu Lake exhibited more stable *I_geo_* values across the lake (Figure 2). The PERI results revealed low ecological risk across all three lakes, with Napahai Lake exhibiting a comparatively higher risk (Figure S5). Specifically, the PERI values for Shudu Lake, Bitahai Lake, and Napahai Lake were 62.7, 65.2, and 76.5, respectively. Furthermore, the $E_{r}^{i}$ results for the target heavy metals (Cu, Zn, As, Cd, Hg, and Pb) in the three plateau lakes indicated that Cd and Hg consistently contributed the most to the potential ecological risk in each lake. Among these, Cd in Napahai Lake exhibited the highest $E_{r}^{i}$ value among the three lakes (Figure 3).

### 3.4. Potential Sources of Potentially Toxic Elements in Surface Sediments of the Plateau Lakes of Shangri-La

The Cu, Zn, and Pb concentrations covaried in Bitahai Lake and Napahai Lake, whereas Hg and As loaded onto relatively independent dimensions. In contrast, Shudu Lake demonstrated a coupled variation pattern among Zn, As, and Cu, while Cd showed only weak correlations with other elements (Figures S6 and S7). The PMF model quantitatively identified four source factors: A Cu–Zn–Pb-dominated ‘traffic/domestic mixed source’(F1), a Cd and Hg dominated ‘agricultural/coal-combustion mixed source’ (F2), an As-indicated ‘agricultural/local point-source factor’ (F3), and a ‘natural parent-material with regional mixed source’ (F4) reflecting lithogenic weathering overprinted by regional atmospheric inputs (Figure S8). Contribution analysis showed that anthropogenic sources (F1–F3) collectively accounted for approximately two-thirds of the total metal inputs across the three lakes, whereas the natural background factor (F4) contributed about one-third (31–36%, Figure 4). The relative source structures were broadly comparable among Bitahai Lake, Napahai Lake, and Shudu Lake. However, the overall metal burdens and risk levels were higher in Napahai Lake than in the other two lakes. Bitahai Lake and Shudu Lake showed relatively lower metal loads, and Shudu Lake was characterized by a distinct Zn–As-enriched factor with secondary Cu.

## 4. Discussion

To investigate the potentially toxic elements pollution status of three typical plateau lakes located in Shangri-La that serve as important wintering habitats for black-necked cranes, Bitahai Lake and Shudu Lake within Potatso National Park, and Napahai Lake outside the National Park, we determined the concentrations of six heavy metal elements in the sediments and systematically assessed their contamination levels and potential ecological risks. To the best of our knowledge, this is the first study to systematically evaluate the potential ecological risks of heavy metals in the surface sediments of these plateau lakes, which are important wintering habitats for the black-necked crane in Shangri-La.

The three plateau lakes in Shangri-La, which serve as important wintering grounds for the black-necked crane, were generally at an unpolluted to slightly polluted status based on the sediment contamination indices, with Napahai Lake exhibiting the highest potential ecological risk (PERI = 76.5). In contrast, as a key wintering habitat for the black-necked crane, Caohai Wetland has been reported to be under considerable pressure from heavy metal pollution (including As, Cd, Cr, Ni, Pb, Sb, and Zn) [28,29]. During the breeding season, the black-necked crane inhabits wetlands on the Qinghai–Tibet Plateau [10]. To obtain a broader understanding of heavy metal contamination in plateau lake sediments, we compared our results with those reported for 12 plateau lakes on the Qinghai–Tibet Plateau, and the PERI values in this study were relatively lower [47]. Compared with Qinghai Lake, the three plateau lakes in this study exhibited higher Cu concentrations, which may point to regional anthropogenic input in Shangri-La [48]. The differences in heavy metal concentrations among different plateau lakes may arise not only from varying degrees of human activity but also from differences in natural background levels. The divergent trends observed for As and Pb may reflect lake-specific hydrological conditions. The higher As concentration in Napahai Lake may be related to evaporative concentration in this seasonal wetland, whereas the elevated Pb levels in Shudu Lake and Bitahai Lake likely may reflect atmospheric deposition associated with tourism-related activities at higher altitudes [49]. Notably, many protected plateau lakes on the Qinghai-Xizang Plateau still face increasing anthropogenic pressures, including atmospheric transport of pollutants and tourism development. Although the plateau lakes in this study showed relatively low PERI values, they still exhibited regional Cu enrichment and site-specific As/Pb anomalies, which are consistent with the pressures observed in other protected plateau lakes.

Napahai Lake exhibited higher overall metal contaminations and potential ecological risk than Bitahai Lake and Shudu Lake. The spatial distribution of exceedance sites supports a possible influence of surrounding human activities on local heavy metal accumulation. In Napahai Lake, Cd exceedance sites were located near roads and guesthouse areas, whereas the co-occurrence of Cd and As exceedances was found near villages and pasture zones, which may indicate localized inputs such as inflow channels, local discharges, or point-source influences [50]. A similar phenomenon has been reported in river–wetland and urban–wetland systems [51,52]. In Shudu Lake, exceedance sites were spatially associated with roads, a pier, pasture, and inflow-affected shoreline sections, indicating that even under relatively strict protection, localized disturbances may still produce detectable sediment contamination signals. In Bitahai Lake, the sole site with co-occurring Cd and Hg exceedances was located near a pier, which may imply that localized tourist infrastructure or shoreline disturbance could contribute to anomalous enrichment. Notably, the abnormal Hg enrichment at specific sites in Bitahai Lake may result from the combined effects of regional atmospheric input, nearshore disturbance, and favorable low-energy depositional conditions for Hg retention. Similarly, evidence from the protected Dajiuhu Wetland in Shennongjia, China, suggests that Cd can be a sensitive indicator of anthropogenic influence, increasing with intensified human disturbance [52,53,54].

Existing studies have shown that changes in land use around lakes, construction projects, and tourism activities can introduce pollutants via surface runoff and atmospheric deposition [31,47,55]. Our findings suggest that management practices may influence pollution control by regulating shoreline land use, anthropogenic disturbance intensity, and contaminant delivery pathways. For the two strictly managed plateau lakes within Potatso National Park, stricter management regimes may have constrained shoreline construction, road access, tourism-related pressures, and other intensive activities, potentially reducing heavy metal inputs. In contrast, the openly managed Napahai Lake is surrounded by villages, pasture, roads, and tourism facilities, creating a more intricate network of potential external inputs. This situation may promotes the transport of pollutants through surface runoff and hydrological connectivity. However, it should be noted that the three lakes also differ in their geological, hydrological, and environmental characteristics, and the higher contamination levels in Napahai Lake cannot be attributed exclusively to its management system. Other factors, such as catchment geology, hydrological connectivity, water residence time, and natural background levels, may also contribute to the observed differences. Therefore, for strictly managed plateau lakes within Potatso National Park, possible measures include restricting lakeshore development, reducing tourism-related disturbances, and strengthening riparian vegetation and soil and water conservation [56,57]. For the openly managed Napahai Lake, priority could be given to watershed scale source control, key channel management, agricultural non-point source pollution control, and regular monitoring of atmospheric deposition [50,51]. Furthermore, we recommend delineating differentiated shoreline control zones for each lake based on pollution hotspots, land use intensity, and hydrological connectivity, with corresponding monitoring frequencies and management measures formulated accordingly.

However, we acknowledge that this study has some limitations. The available data are insufficient to fully characterize heavy metal exposure and the associated health risks in wintering birds. Moreover, this study did not evaluate the seasonal variation in heavy metal concentrations. Therefore, the results reflect a snapshot at a single time point rather than a long-term trend. Future studies should integrate heavy metal measurements in black-necked crane feces with those in environmental samples, including water, and potential food sources, such as grains, potatoes, and invertebrates. Such integrated investigations would help identify potential exposure sources and pathways and support a more comprehensive assessment of health risks associated with habitat pollution. Despite these limitations, our study provides a useful reference for habitat management and scientific conservation of wintering birds such as the black-necked crane, and also lays a solid foundation for the management of similar plateau lake ecosystems.

## 5. Conclusions

This first comprehensive assessment of heavy metal pollution in black-necked crane wintering habitats on the Yunnan Plateau reveals that the three representative plateau lakes in Shangri-La remain at an unpolluted to slightly polluted status with low ecological risk (PERI: 62.7–76.5), with Napahai Lake exhibiting the highest risk (PERI = 76.5) due to less restrictive management. Source apportionment identifies traffic/domestic emissions (Cu-Zn-Pb), agricultural/combustion sources (Cd-Hg), and localized inputs (As) as the main anthropogenic contributors, indicating that surrounding human activities significantly influence local heavy metal accumulation. Our findings provide a critical scientific reference for the habitat management and conservation of the black-necked crane and other migratory waterbirds in plateau lake ecosystems.

## Figures and Tables

**Figure 1:**
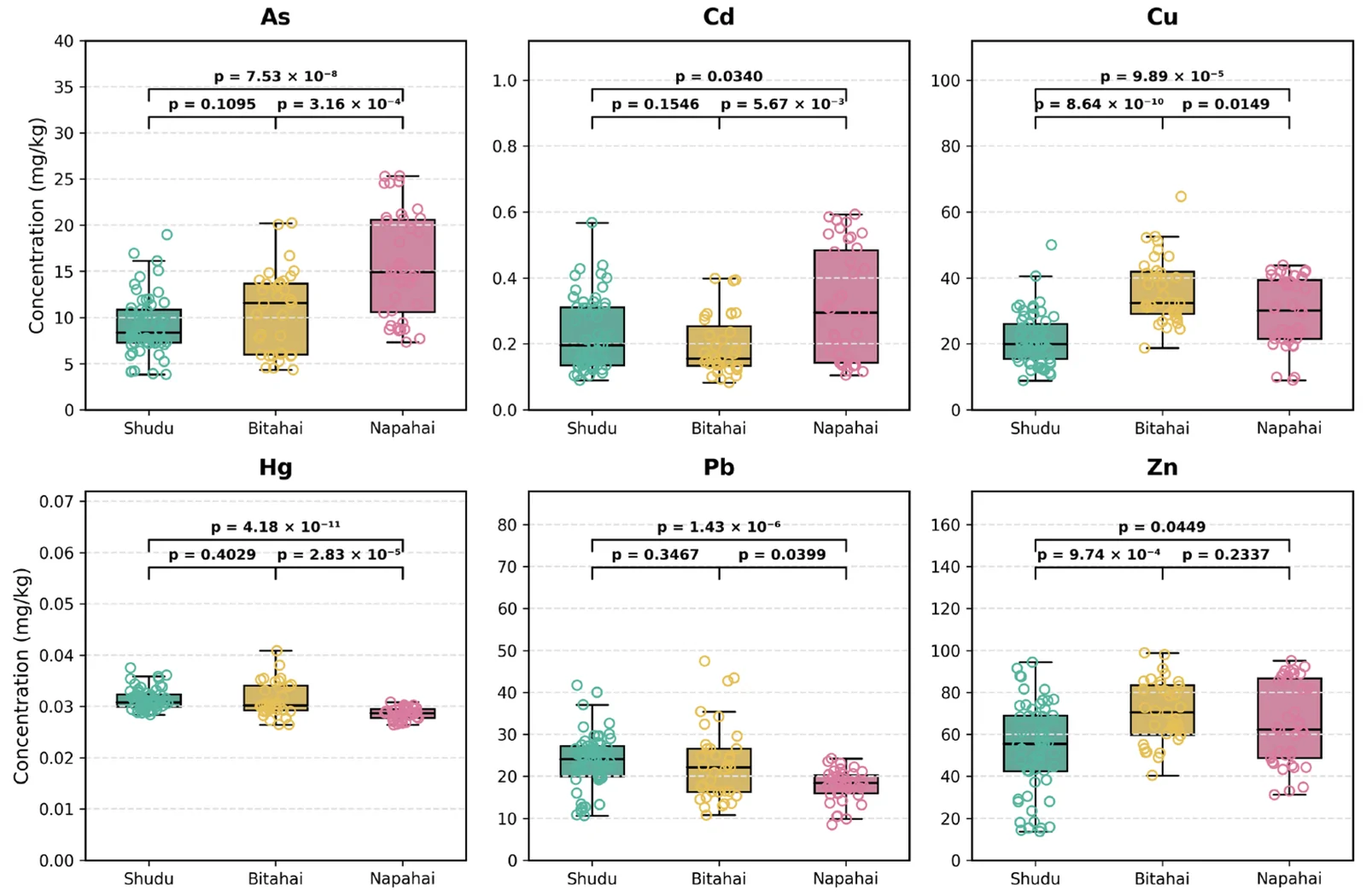
Boxplots of Cu, Zn, As, Cd, Hg and Pb concentrations in surface sediments of Shudu Lake, Bitahai Lake, and Napahai Lake. The y-axis represents the concentration of heavy metals (μg/g). Hollow circles represent individual sample data points.

**Figure 2:**
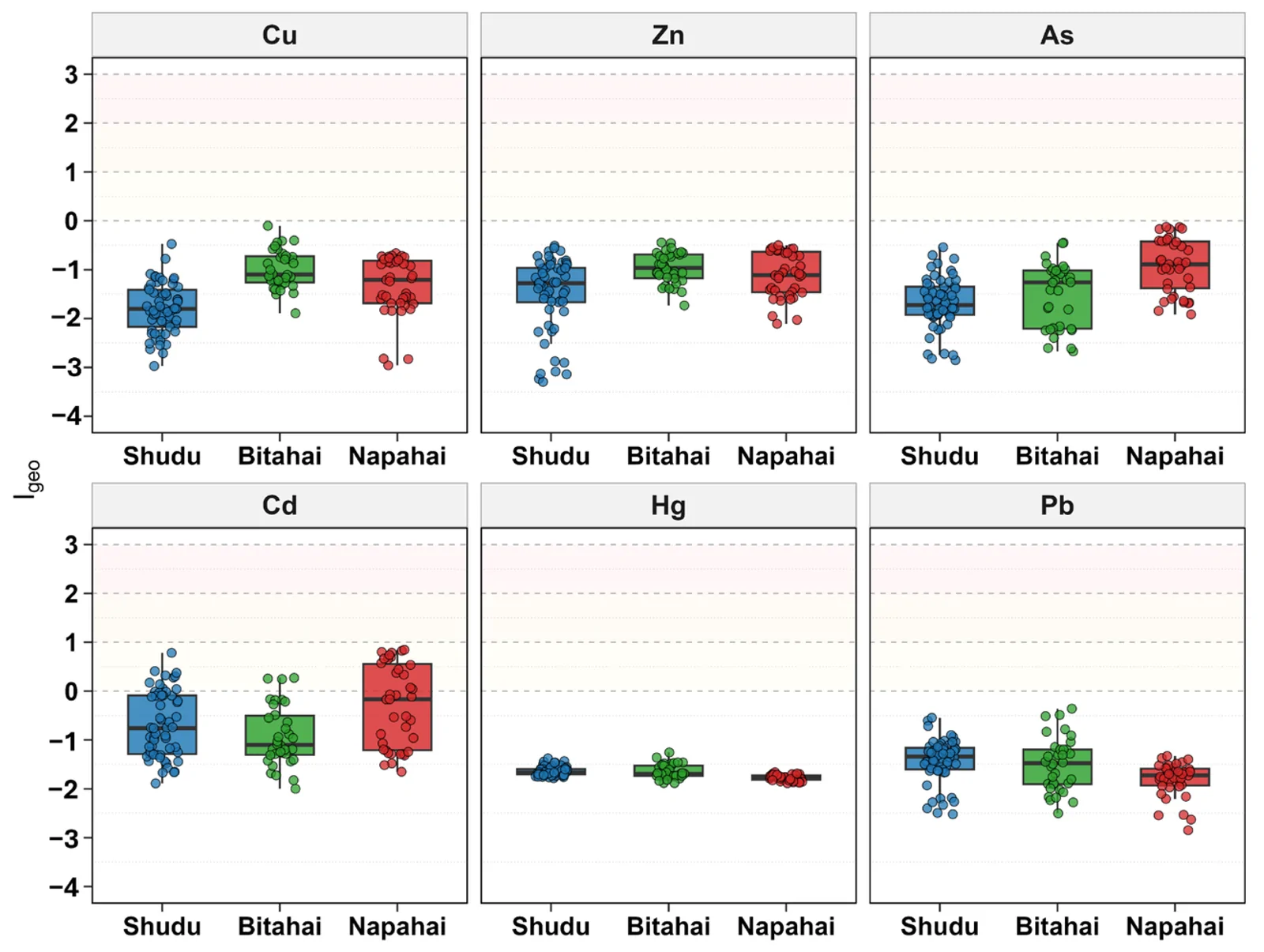
*I_geo_* values of black-necked crane habitats from plateau lakes of Shangri-La.

**Figure 3:**
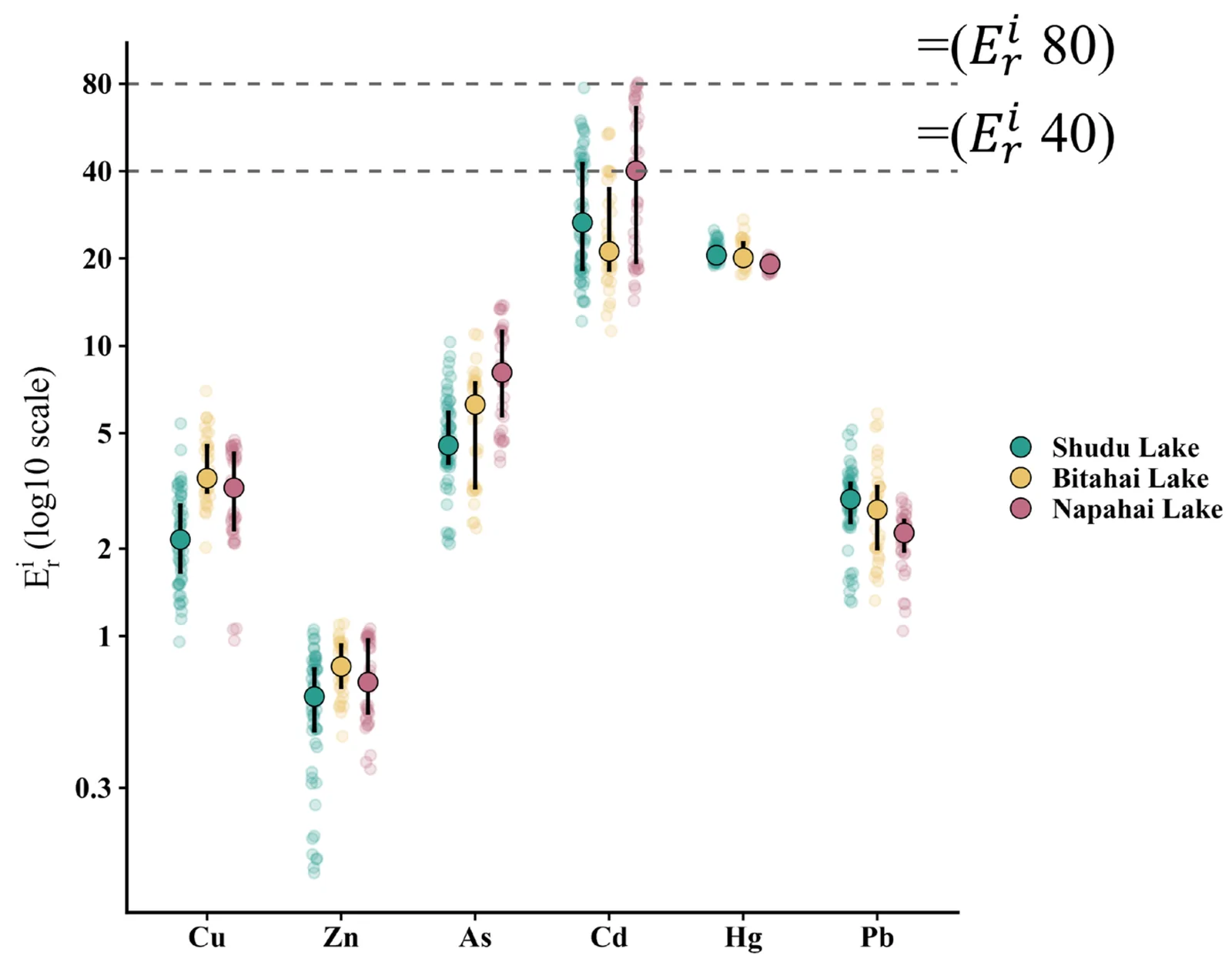
Distribution of $E_{r}^{i}$ values of black-necked crane habitats from plateau lakes of Shangri-La.

**Figure 4:**
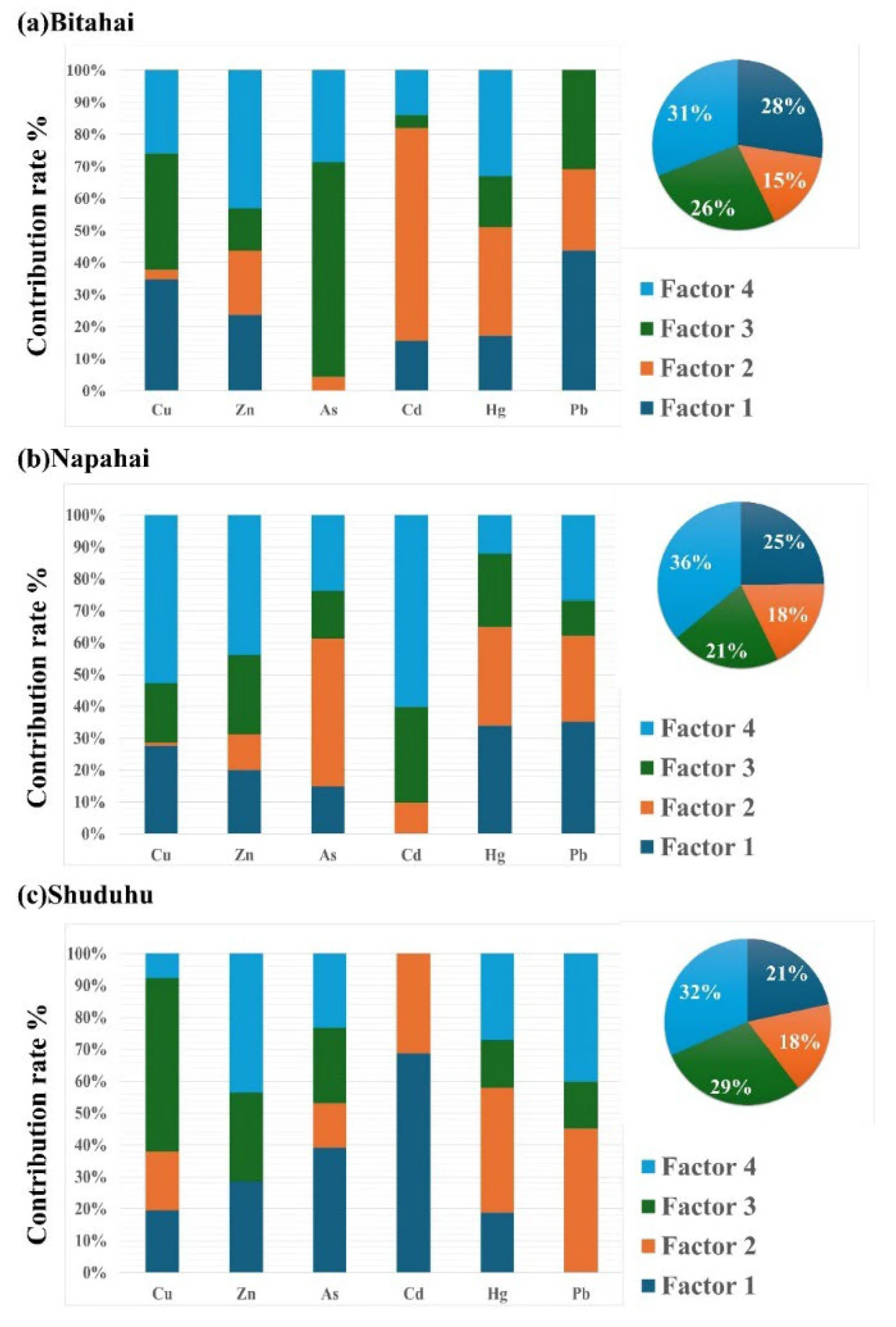
Contribution ratios of sediment heavy-metal pollution sources based on PMF model analysis.

**Table 1:** Grading standard of ground accumulation index.

Range	Pollution Level	Degree of Contamination
*I_geo_* ≤ 0	0	Unpolluted
0 < *I_geo_* ≤ 1	1	Very slightly polluted
1 *< I_geo_ ≤* 2	2	Slightly polluted
2 *< I_geo_ ≤* 3	3	Moderately polluted
3 *< I_geo_ ≤* 4	4	Moderately to heavily polluted
4 *< I_geo_ ≤* 5	5	Heavily polluted
*I_geo_* > 5	6	Severely polluted

## Data Availability

Data will be made available on request.

## Supplementary Materials

The following supporting information can be downloaded at: https://www.mdpi.com/article/10.3390/ani16193012/s1.
