# Supplementary material for: Potentially Toxic Elements in Surface Sediments of Black-Necked Crane Habitats: A Case Study of Plateau Lakes in Shangri-La, Yunnan, China
Source: Animals (Basel). 2026 Sep 24;16(19):3012. doi: 10.3390/ani16193012 (PMC13663418; doi:10.3390/ani16193012)
Supplement: Supplementary file 1 [file animals-16-03012-s001.zip › Supplementary Materials.pdf]

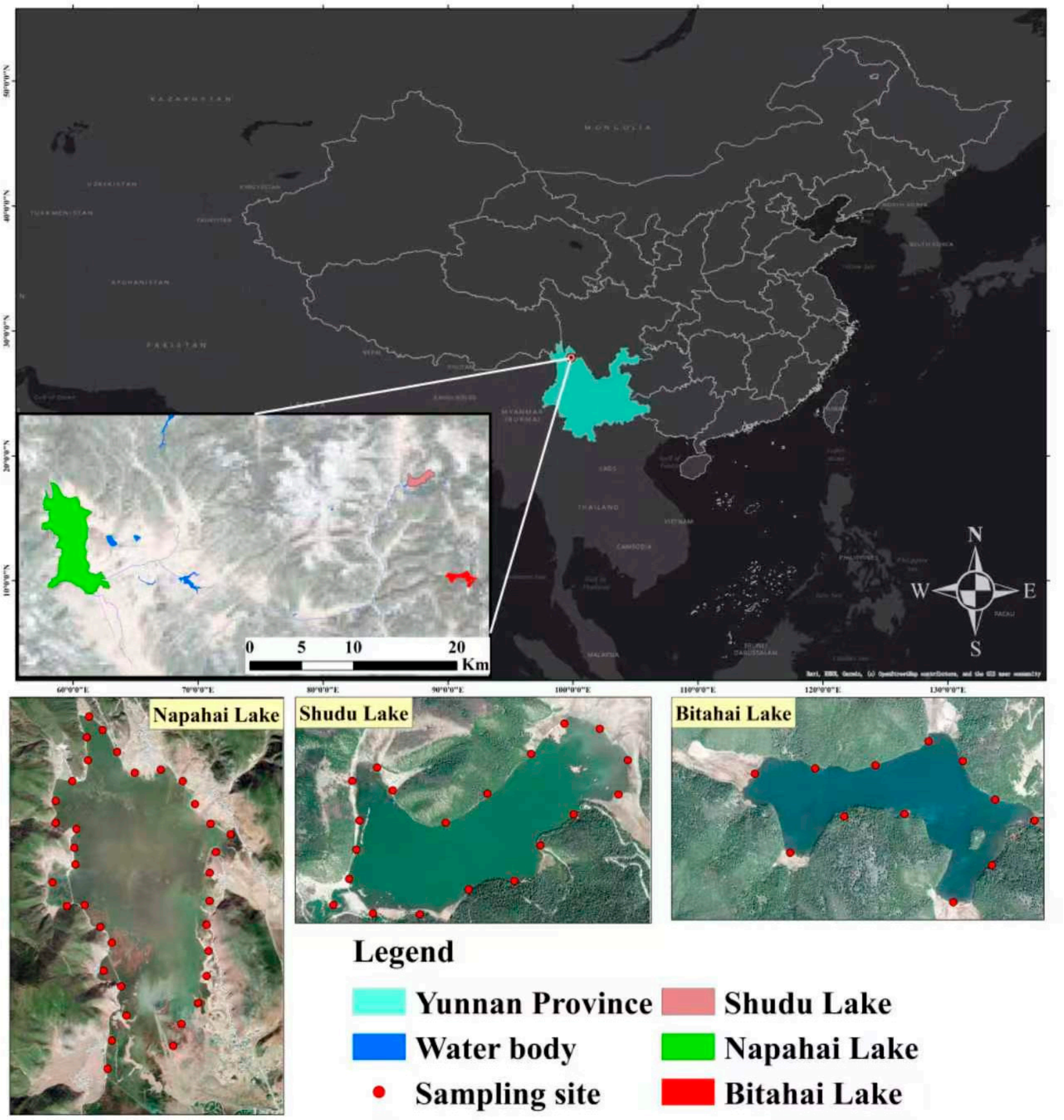

Figure S1 Location of the study area and sampling sites in Shudu Lake, Bitahai Lake, and Napahai Lake

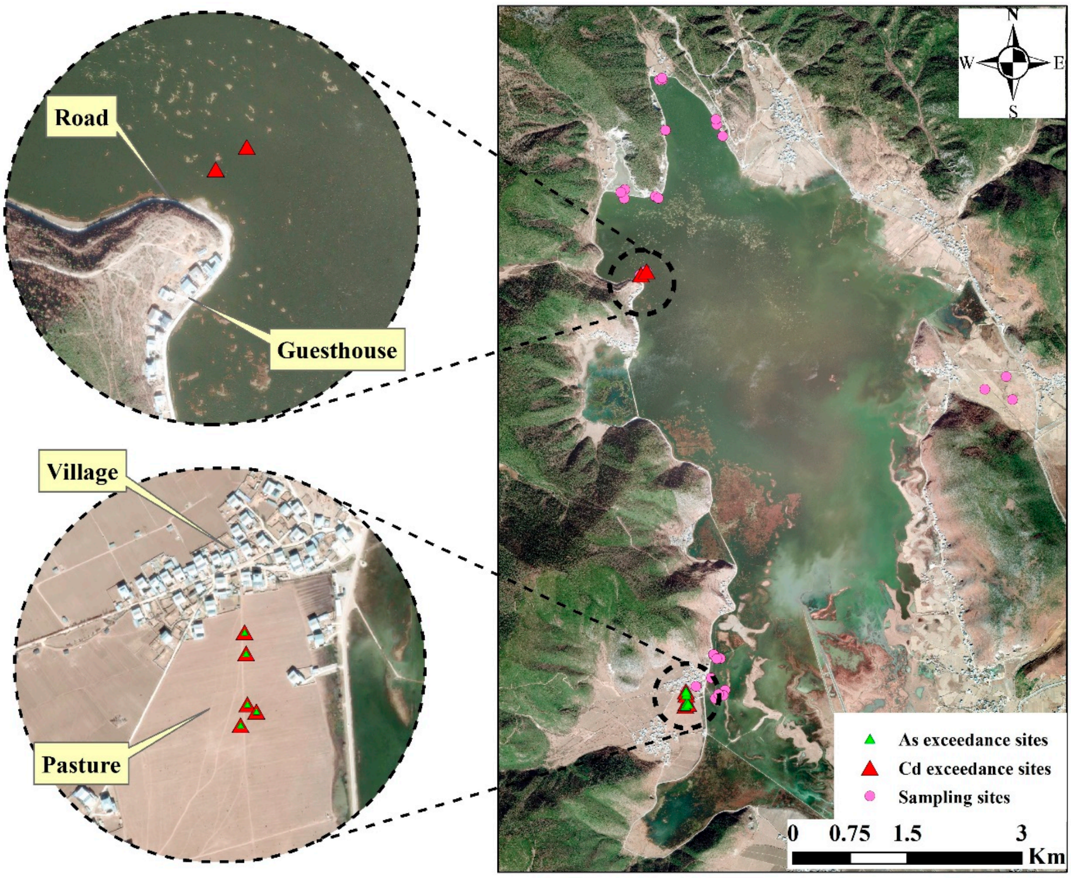

Figure S2 Spatial distribution of Cd and As exceedance sites in Napahai Lake and their surrounding human activity features

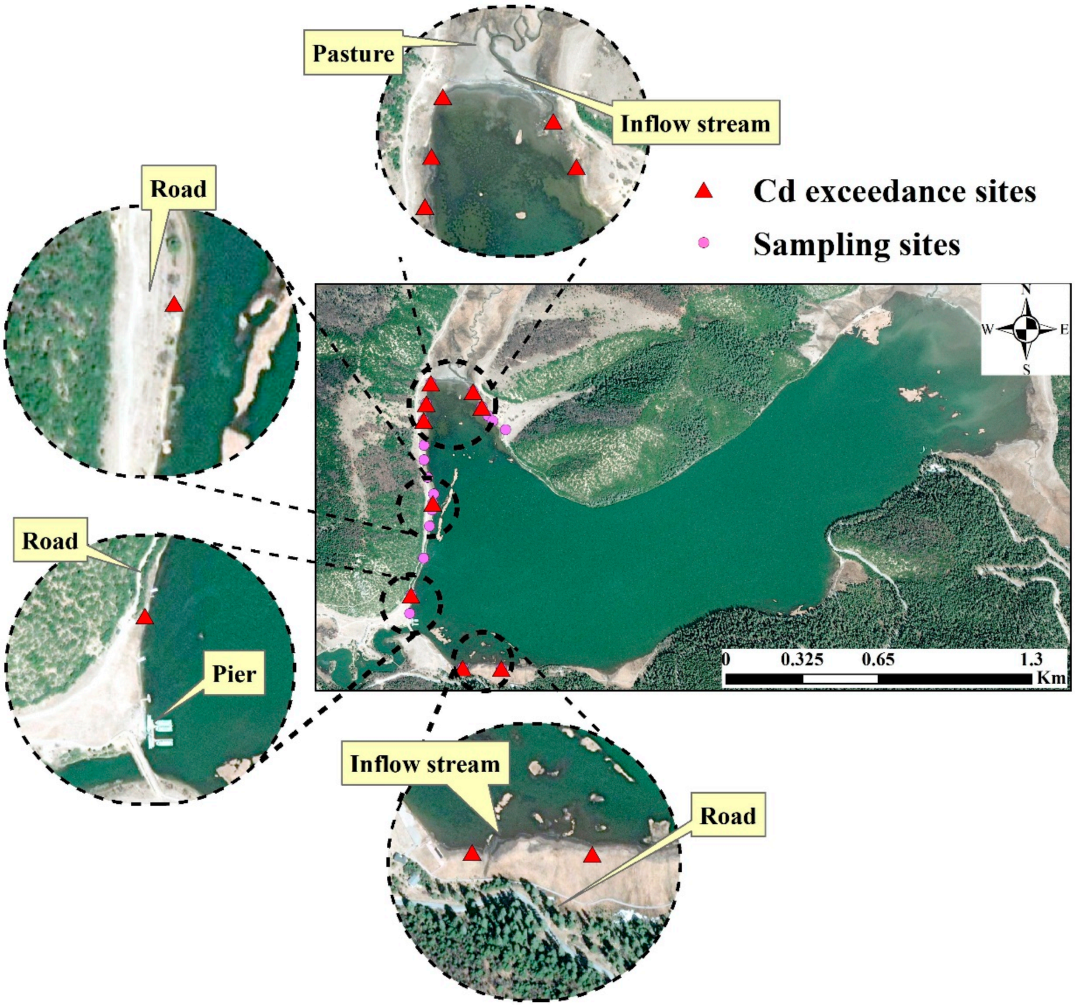

Figure S3 Spatial distribution of Cd exceedance sites in Shudu Lake and nearby potential disturbance features

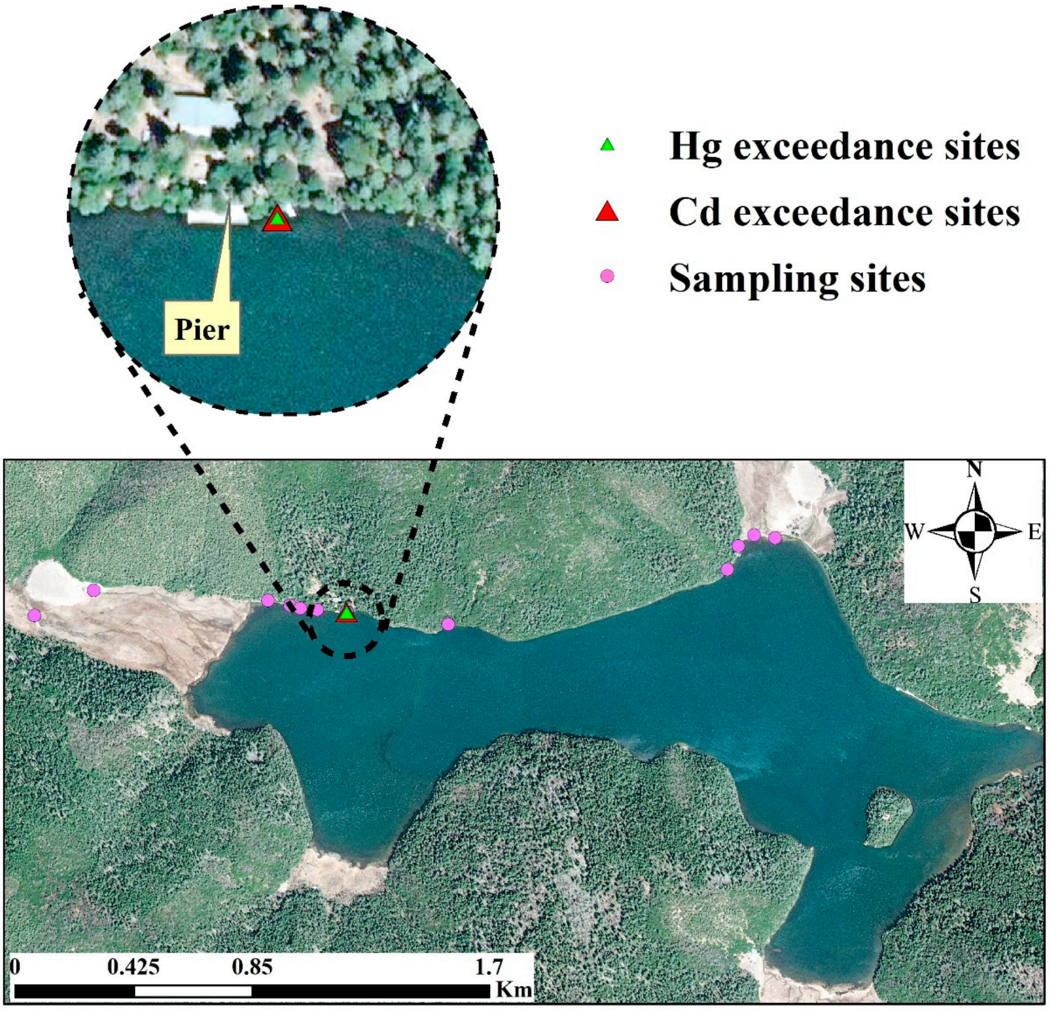

Figure S4 Spatial distribution of Cd and Hg exceedance sites in Bitahai Lake and nearby pier-related disturbance

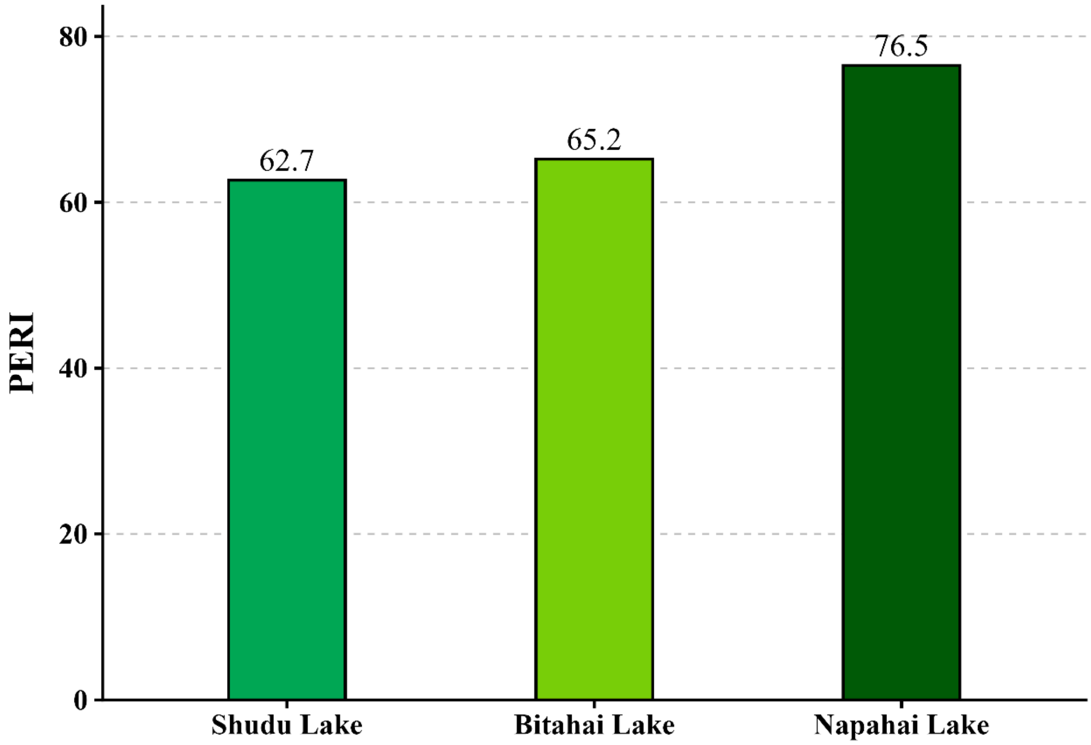

Figure S5 Mean potential PERI values for surface sediments in Shudu Lake, Bitahai Lake, and Napahai Lake. Bars represent the mean PERI values across sampling sites within each lake, and numbers above the bars indicate the corresponding means.

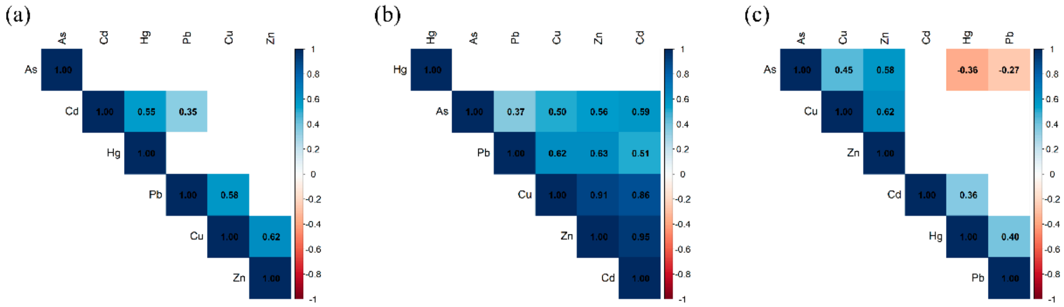

Figure S6 Spearman's rank correlation heatmaps for heavy metals concentrations in sediments of (a) Bitahai Lake, (b) Napahai Lake, (c) Shudu Lake. Numbers within the cells indicate Spearman's correlation coefficients (ρ). Blue and red represent positive and negative correlations, respectively, with darker colors indicating stronger correlations. The color scale ranges from −1 to 1.

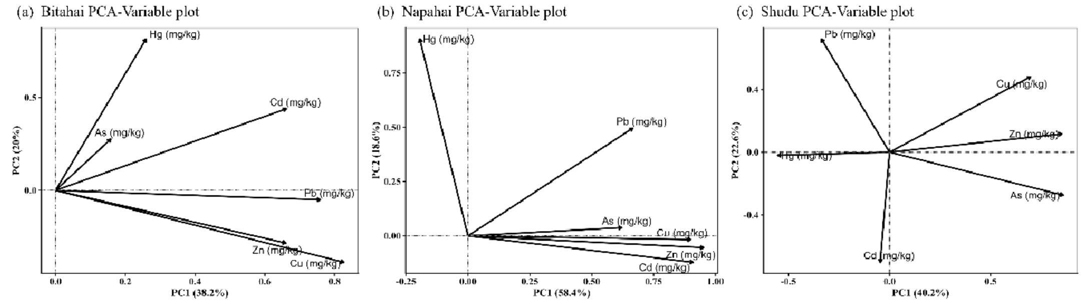

Figure S7 PCA plots for heavy metal concentrations in surface sediments of (a) Bitahai Lake, (b) Napahai Lake, and (c) Shudu Lake. Arrows represent the loading vectors of individual elements on the first two principal components (PC1 and PC2). Percentages in the axis labels indicate the proportion of total variance explained by each principal component. Dashed lines indicate zero loadings on the corresponding axes.

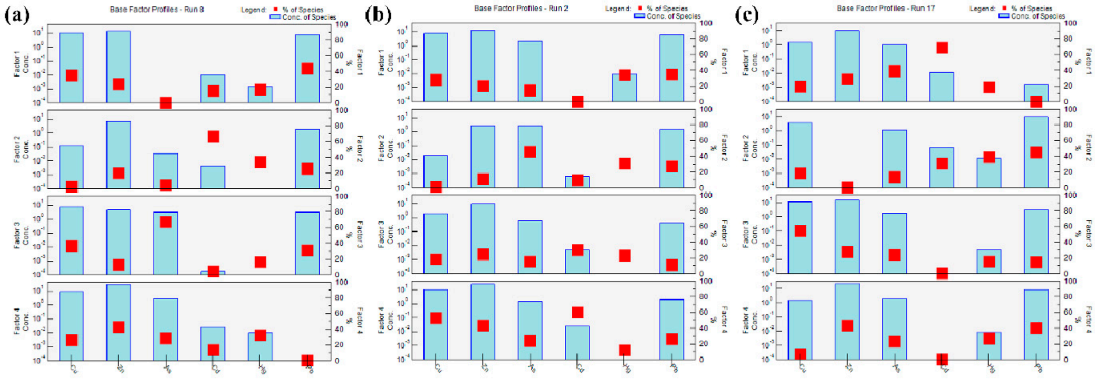

Figure S8 Factor profiles (source fingerprints) of heavy metals resolved by the PMF model (a) Bitahai Lake, (b) Napahai Lake, (c) Shudu Lake
